# Environmental Complexity and Reduced Stocking Density Promote Positive Behavioral Outcomes in Broiler Chickens

**DOI:** 10.3390/ani13132074

**Published:** 2023-06-23

**Authors:** Lauren Evans, George C. Brooks, Mallory G. Anderson, Andrew M. Campbell, Leonie Jacobs

**Affiliations:** 1 Virginia Tech, School of Animal Sciences, 175 West Campus Drive, Blacksburg, VA 24061, USA; lauren00@vt.edu (L.E.); mga5@g.clemson.edu (M.G.A.); drewc1@vt.edu (A.M.C.); 2 Virginia Tech, Department of Fish and Wildlife Conservation, 310 West Campus Drive, Blacksburg, VA 24061, USA; boa10gb@vt.edu

**Keywords:** animal welfare, broiler chicken, behavior, environmental enrichment, ethology, stocking density

## Abstract

**Simple Summary:**

Broiler chickens are usually housed in high-density, relatively barren conditions that limit their opportunities to express natural behaviors and be active. This study aimed to investigate the effects of complex environments and stocking densities on broiler behavior. The frequency and duration of broiler chicken behaviors, such as preening, dustbathing, foraging, and play were recorded at different time points when birds were 2, 4, and 7 weeks old. We found that environmental complexity positively influenced foraging, locomotion, preening, and overall activity. However, dustbathing and play were not affected by the complexity of the environment. High stocking density resulted in more frequent but not longer activity overall, which suggests disturbances. In addition, high density resulted in less frequent foraging, drinking, and eating. As chickens aged, their activity levels reduced, and they showed potential signs of frustration. However, the benefits of environmental complexity and low density persisted. Overall, providing a complex environment with a low stocking density can enhance broiler chicken welfare by stimulating their natural behaviors.

**Abstract:**

The objective was to evaluate the impacts of a complex environment and stocking density on Ross 708 broiler chicken behaviors. Eight pens contained either high complexity (HC) or low complexity (LC) environments, and high (HD) or low (LD) density. Through focal-animal sampling, the frequency and duration of behaviors were recorded continuously for 5 min at two timepoints for one day in weeks 2, 4, and 7. Birds were active for 30% of the observed time, with birds showing more activity in HC compared with LC. Birds in HC pens spent more time preening and foraging than birds in LC pens, which was interpreted as a positive outcome. Dustbathing and play were not impacted by complexity, possibly due to the observation method. Birds were more frequently active at HD compared with LD, but did not spend more time being active, suggesting disturbances. Birds foraged, drank, and ate less frequently in HD compared with LD, presumably because birds had more difficulty accessing resources. Activity and active behaviors reduced as birds aged, while preening frequency increased, possibly due to frustration, but this was not confirmed. Perching was unaffected by age, showing a persistent motivation to perform the behavior. Our results indicate that a complex environment provides positive stimulation for foraging, locomotion, preening, and overall activity. Despite reduced activity, many benefits of the tested environmental complexity and low density persisted as birds aged.

## 1. Introduction

The welfare of production animals strongly relies on their ability to perform natural behaviors [1]. When animals are unable to express highly motivated behaviors, it can be detrimental to their affective state [2]. Affective states are prolonged mood states influenced by the sum of life experiences and can range from positive to negative [1]. Providing opportunities for behavioral expression that facilitate a positive affective state is therefore an important component of animal welfare [1].

The natural behavior of broiler chickens includes eating, drinking, sleeping, preening, running, jumping, scratching, ground pecking, wing flapping, stretching, dust bathing, playing, fighting, and vocalizing [3]. However, broiler chickens are genetically selected for fast growth [4], which is often associated with decreased physical ability and health, more specifically leg issues, muscle myopathies, and pulmonary and cardiovascular issues [5,6,7]. These physical defects can reduce a bird’s ability to perform certain behaviors and impact their behavioral repertoire [7,8]. In line, inactivity in fast-growing broiler chickens ranges from 61 to 76% and can be as high as 86% when birds experience lameness [9,10,11]. Broilers that experience lameness can have difficulty reaching feed and water [9,12]. In addition, the expression of natural behaviors such as eating can be modified, with lame broilers tending to lie down to eat about half of the time, compared with standing while eating in broilers without gait impairment [13]. 

Broiler chickens are typically housed in relatively barren conditions [6]. Although they have food, water, litter, and conspecifics, other resources are often lacking. Furthermore, commercial houses are monotonously designed, in part to stimulate the homogeneous distribution of the flock. These conditions minimize production costs and maximize space availability, and therefore potential profitability [6]. Controllability, i.e., the choice to seek stimulation from a variety of resources, is lacking in these conditions. Controllability (choice) is long recognized as an important aspect of good animal welfare, or “physiology and behavior” as described by [14], and barren, monotonous conditions limit birds’ ability to choose and express a range of behaviors. Akin to their jungle fowl ancestors, broilers are motivated to perch or rest on elevated surfaces [15], dust bathe [16,17], and show food seeking behavior (foraging) [16,18,19]. Providing environmental enrichment stimulates more frequent and a greater variety of natural behaviors in broiler chickens and can result in improved animal health and affective states [20,21,22,23,24,25,26]. For instance, access to perches or barriers, or changing lighting variation can increase activity in broilers [27,28,29,30].

The high stocking densities at which broiler chickens are typically housed is expected to impact the expression of natural behaviors. The maximum commercial broiler chicken stocking densities for the United States range between 31.7 kg/m^2^ and 43.9 kg/m^2^ depending on the final target liveweight [31]. In the European Union, legislation prescribes a maximum stocking density of 33 kg/m^2^, yet if mortality targets are met, the maximum allowable stocking density is 42 kg/m^2^ [32]. Birds housed at high stocking densities spend less time at the feeders and more time at the water lines compared with birds that were housed at lower densities [33]. Furthermore, higher stocking densities resulted in decreased opportunities for undisturbed rest [34,35], and more pecking and fighting compared with lower densities in slow-growing broilers [36]. Importantly, even though bird numbers remain constant over time or are reduced due to thinning, mortality, and culls, stocking density increases in terms of kgs per m^2^ as birds grow.

The natural behavior for a broiler chicken changes with age. For example, broiler chickens become less active as they age [19,37,38], with play [39], perching [40], foraging, and locomotion decreasing with age [19]. Such changes, however, may depend on growth rates and housing environments (e.g., [8]). For example, at high stocking densities, access to resources may become increasingly constrained by space and competition as birds grow. Similarly, string enrichment interactions peak at 3 weeks of age, with authors suggesting that physical limitations could be one of the reasons that strings are not frequently used later in life [41]. Thus, it is necessary to characterize behavioral repertoires across the lifetime of birds to fully understand the impact of housing environment on bird welfare. 

Here, we evaluate broiler chickens’ behavioral expression in response to environmental complexity and stocking density. Our objectives were to (1) determine whether environmental complexity can help to promote natural behaviors, (2) evaluate the degree to which stocking density limits natural behaviors, and (3) quantify both natural and environmentally mediated changes in behavioral repertoires as birds age. We hypothesized that the low stocking density and high environmental complexity treatments would result in a higher total duration of positive behaviors such as perching, foraging, dustbathing, and exploring, compared with the high stocking density and low environmental complexity treatments. We further hypothesized that the low-density and high-complexity treatment would result in more sustained activity compared with the high-density and low-complexity treatments as birds aged. This could imply that both space provision and access to a complex environment result in a higher level of welfare for fast-growing broiler chickens compared with contrasting conditions.

## 2. Materials and Methods

This experimental study was conducted between February and March of 2020 and was approved by the Institutional Animal Care and Use Committee (IACUC) of Virginia Tech (protocol 19-175). 

### 2.1. Animals and Housing

For this experiment, 1080 Ross 708 male broiler chicks (Marek’s vaccinated) were transported from a commercial hatchery (Elizabethtown, PA, USA) at day 0 to the Virginia Tech poultry facility where they were housed until day 50 of age as this is in line with the average processing age in the Unites States broiler chicken industry [42]. The birds were randomly allocated in one of four treatment groups. Two environmental complexity treatments and two stocking density treatments were tested in a 2 × 2 factorial approach. Chicks were housed in 8 pens, which was a subset of a total of 12 pens as part of a larger study [20,43]. Each treatment had two replicates. The pens (14.5 m^2^) contained pine wood shavings (~10 cm deep), three water lines with three nipple drinkers each, and four galvanized tube feeders. All birds had ad libitum access to water and commercial broiler chicken feed (starter day 0–14, grower day 15–28, and finisher day 29–50). Pens contained heat lamps and 24 h light in the first seven days. Due to a technical issue, lights were on continuously on days 8–14. After the second week, lighting was on for 18 h (light intensity of approximately 15 lx during light hours) followed by 6 h of continuous darkness. House temperature was gradually lowered from 35 °C on day 1 to 21 °C on day 21, and was consistent until day 50. We provided a therapeutic dose of antibiotics via the water lines to all birds from day 33 to 40 in response to a respiratory disease (infectious bronchitis) outbreak that resulted in an increased cull and mortality rate (approximately 3.6% total mortality) after pathogen exposure.

### 2.2. Environmental Complexity

Four pens contained a high-complexity (HC) treatment, and four pens a low-complexity (LC) treatment. Each pen was roughly separated into four spaces. In the HC pens, the spaces were targeted at different functions, increasing the birds’ ability to make choices based on their motivation. Space 1 (3 m^2^) targeted “feeding” and contained four commercial feeders and one third of a PECKstone^TM^ (Proteka, Inc., Lucknow, ON, Canada) broken into smaller pieces. Space 2 (3 m^2^) targeted “comfort” and contained a wooden dust bath (180 cm L × 91 cm W × 10 cm H) filled with playground sand. Space 3 (3 m^2^) targeted “resting” and contained three PVC perches (183 cm L × 31 cm W × 9 cm H, with 1.9 cm diameter pipes) as shown in [43]. Space 4 (4.3 m^2^) targeted “exploration” and contained temporary enrichments. Six temporary enrichments were randomly paired, combining nutritional and occupational enrichment. Each HC pen received eight hanging bundles of white string, four metal balls (20.3 cm diameter) filled with alfalfa hay, four treat dispenser balls (7.6 cm diameter) filled with whole-grain oats, four colored balls (5.8 cm diameter), four classic Kong toys (5.6 cm diameter; Kong Company LLC, Golden, CO, USA) filled with lettuce, and laser light interactions for 5 min twice a day. These enrichment sets were rotated every three days according to a randomized schedule to maintain novelty, resulting in birds having access to a single pair of enrichments at a given time. The random order of the three enrichment sets was repeated throughout the production period, meaning that the birds received the same set of enrichments after six days of exposure to the other enrichments (i.e., set 1, set, 2, set 3, set 1, …).

In the LC pens, the configuration of the four spaces was the same as in the HC pens, yet no enrichments were provided. The feeders were distributed across spaces 1–3, with two feeders in space 1, one feeder in space 2, and one feeder in space 3. Space 4 contained litter but no other resources.

### 2.3. Stocking Density

Half the birds were stocked at a high density (HD) with 180 birds per pen (12.4 birds/m^2^; 4 pens), to approach commercial broiler chicken production standards. The other half were stocked at a low density (LD) with 90 birds per pen (6.2 birds/m^2^; 4 pens), about half the commercial standard to ensure a large contrast between the two treatments. Stocking density was 6.2 kg/m^2^ for HD and 2.9 kg/m^2^ for LD pens on day 15, 18.9 kg/m^2^ for HD and 9.8 kg/m^2^ for LD pens on day 29, and 42.0 kg/m^2^ for HD and 23.8 kg/m^2^ for LD pens on day 50. We did not incorporate the space occupied by enrichments when calculating the stocking density, meaning that birds in HC environments had slightly less space than birds in LC pens.

### 2.4. Measurements 

Behaviors were recorded using two cameras per pen (IP Bullet camera FLPB133F, FLIR Systems Inc., Wilsonville, OR, USA, now available through Lorex Technology Inc., Markham, ON, Canada). Spaces 1 and 2, and the majority of space 4 were best visible on videos from one camera. Space 3 and the back of space 4 were best visible on videos from the second camera. For one pen (LC/HD), only one camera was available due to a technical problem with the second camera. We were unable to code behaviors in Space 3 for the pen with one camera. Videos were recorded one day a week at 8 AM and 4 PM in week 2, 4, and 7 of age (12 February (Week 2), 25 February (Week 4), and 17 March (Week 7)). 

Behavioral frequencies and durations (Table 1) were coded from videos using Behavioral Observation Research Interactive Software (BORIS; [44]).

We used 5 min continuous focal animal sampling, selecting 12 birds per pen time point (8 AM and 4 PM) per week. Timepoints were selected to ensure there was no human disruption during video recording, to obtain a better representation of the behavioral repertoire rather than just during one timepoint, and because these are generally considered active time periods for chickens. For bird selection, each space was roughly divided into three sections (front, middle, back). An arbitrarily picked bird within a section, within a space (1–4) was then observed for 5 min. Thereafter, the video was rewound and the next bird was selected in a different section within the same space. Three birds per space were observed per pen, resulting in a total of 12 birds per pen per time point. For the pen with one camera, only 9 birds were observed as space 3 was not fully visible. 

Observation order of space (1–4) and section within space (front, middle, back) were randomized for each pen to avoid unintended selection bias. Birds were not marked for identification, but our selection method ensured that 12 individuals were observed at a single time point. The two videos from a single pen at a single time point and date were played simultaneously to ensure that the focal bird was visible throughout the recording. 

Four observers coded the birds’ behaviors. The observers could not be blinded for treatments, but were unaware of the research hypotheses. The inter-observer reliability using the Cronbach’s Alpha scoring for all four observers equaled 92.5%. Per time point (8 AM and 4 PM), 480 min were observed, resulting in 960 min of data collection per week, and 2880 min in total across pens and all three weeks of observation. 

### 2.5. Statistical Analyses

Entries in BORIS were exported to Microsoft Excel (Microsoft Corporation, Redmond, WA, USA) and restructured prior to analysis. All active behaviors (all but resting and sitting) were summed to obtain an “active” behavior category, excluding other and out of view (Table 1). We used hurdle models to analyze counts of chicken behaviors [46,47,48]. The models constitute (1) a binomial generalized linear model, with logit link, for whether any behaviors were observed within a single 5 min observation for an individual bird, and (2) a zero-truncated negative binomial model for individuals with observed behaviors. For both components, we included stocking density, environmental complexity, and the week as predictors. We compared models with one, two, and all three predictors, as well as exploring possible interactions between each of the covariates. Models were compared in an AIC framework and ranked by Akaike weights [49]. Model fit was evaluated with rootograms [50], and predictive performance was assessed using a generalization of the receiver operating characteristic curve (AUC) [51]. Confidence intervals (95% CIs) and *p*-values for parameter estimates were computed using the Wald approximation [52]. 

For the duration of behaviors, we performed the same model ranking approach using zero-or-one-inflated beta regression (ZOIB [53]) to model the proportion of time spent engaging in a behavior during the observation window. As with the hurdle models, ZOIB models include two components that are modelled separately to accommodate excess zeros, and the two components are not constrained to have the same suite of predictors. As before, we included stocking density, environmental complexity, and the week as predictors, and compared models with one, two, and all three predictors, and their interactions. All analysis was conducted in R (R Core Team, 2022), using the countreg and gamlss packages [54]. Pairwise comparisons were performed and summary statistics were calculated using the packages pROC [55] and emmeans [56]. Figures were generated with the packages emmeans [56] and ggplot2 [57].

## 3. Results

Over the course of the study, the behavioral repertoire of 484 birds was documented. A total of 2715 individual counts of behaviors were observed across 420 birds exhibiting active behaviors (Table 2 and Table 3). On average, birds were active for 30% of the time spent under observation, but this varied considerably by individual, treatment, and the week of study (Table 4). Both in terms of the number of birds exhibiting the behavior and the total count of observations, locomotion was the most common active behavior (Table 2 and Table 3). In contrast, dustbathing was observed in the fewest number of birds, and enrichment interactions were observed the fewest number of times overall.

### 3.1. Overall Activity

**Figure 1:**
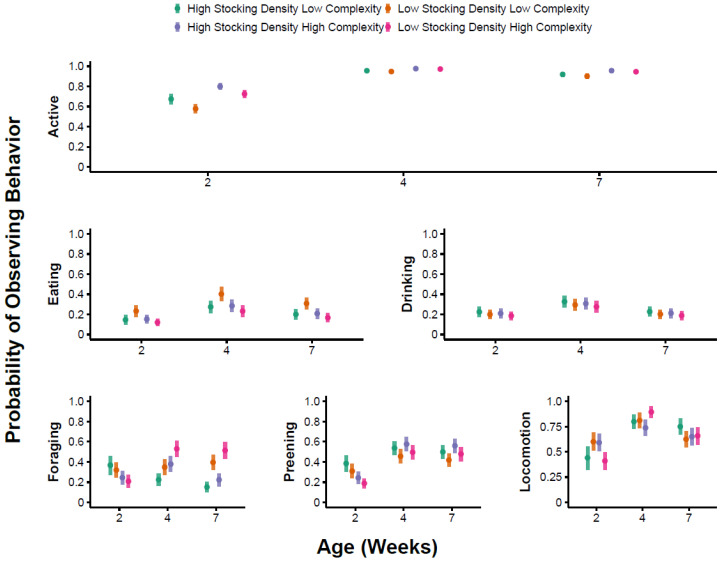
The probability of observing behaviors of interest (0.5 would represent a 50% chance the behavior is observed) for each level of stocking density (high or low density), complexity treatment (high or low complexity), and bird age (2, 4, and 7 weeks of age). Active behavior is the combination of all observed behaviors except for sitting and resting. Whiskers represent 95% confidence intervals.

**Figure 2:**
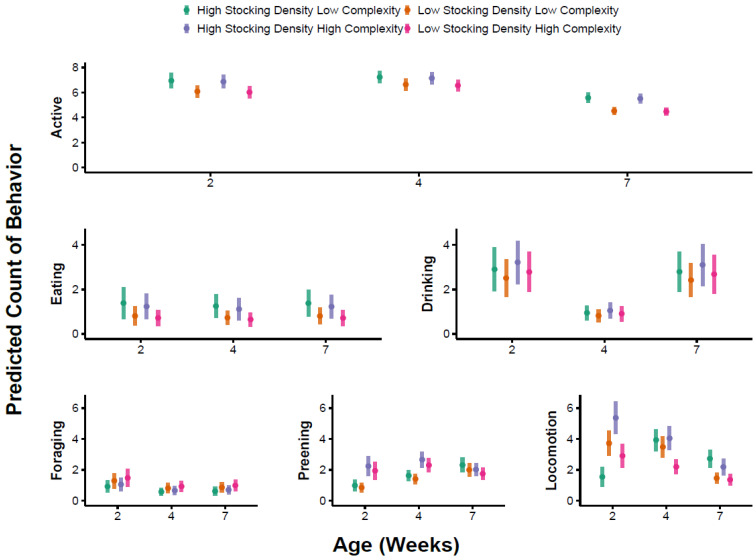
Predicted counts of behaviors (per bird, per five-minute observation) for each level of stocking density (high or low density), complexity treatment (high or low complexity), and bird age (2, 4, and 7 weeks of age), with 1 representing that the behavior of interest is predicted to occur once in an individual bird within a 5-min observation period. Predictions are conditional on a behavior being observed at least once during the 5-min observation period. The whiskers represent 95% confidence intervals. Active behavior is the combination of all observed behaviors except for sitting and resting.

**Figure 3:**
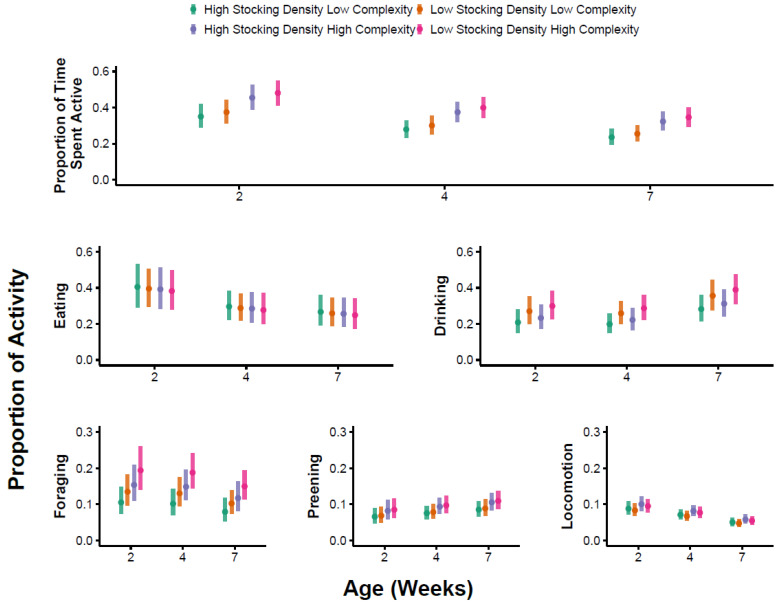
The proportion of time spent performing behaviors of interest as a function of stocking density (high or low density), complexity (high or low complexity), and bird age (2, 4, and 7 weeks of age). The whiskers represent 95% confidence intervals. A proportion of 0.5 would represent that we predict 50% of the observed time is spent performing the behavior of interest. Active behavior is the combination of all observed behaviors except for sitting and resting. Predictions are conditional on a behavior being observed at least once during the 5-min observation period.

The best supported model for the probability of broilers showing any active behavior contained age and environmental complexity as predictors (Table S1). Specifically, birds were more likely to exhibit active behavior in HC environments compared with LC environments (*p* = 0.02, Table S1, Figure 1). Birds were more likely to exhibit active behavior in weeks 4 and 7 compared with week 2 (*p* < 0.001, Table S1, Figure 1). The probability of birds being active did not differ between weeks 4 and 7 (Tukey’s, *p* = 0.38). For counts of active behavior, the best supported model included the effect of week and stocking density (Table S1). Birds exhibited lower counts of active behavior in week 7 compared with weeks 2 (*p* = 0.01) and 4 (Tukey’s, *p* = 0.002), but there was no difference in counts of active behavior between weeks 2 and 4 (*p* = 0.54, Table S1). Birds also exhibited lower counts of active behavior at LD compared with the HD treatments, but this effect was marginal (*p* = 0.08, Table S1, Figure 2). The best supported model for the proportion of time spent active included age and environmental complexity (Table S1). Birds that were active (*n* = 420, 87%) were so for a larger proportion of time in the HC treatments compared with the LC treatments (*p* < 0.001, Table S1, Figure 3). Furthermore, a greater proportion of time was spent active in week 2 compared with week 4 (*p* = 0.02) and week 7 (*p* < 0.001, Table S1, Figure 3). No difference was observed in the proportion of time spent active at week 4 compared with week 7 (Tukey’s, *p* = 0.17). 

### 3.2. Preening

The best supported model for the probability of preening behavior included the week as a predictor (Table S1). Specifically, birds were more likely to be observed preening in week 4 compared with week 2 (*p* < 0.001, Table S1, Figure 1) and in week 7 compared with week 2 (*p* < 0.001, Table S1, Figure 1). The probability of observing preening behavior did not differ between weeks 4 and 7 (Tukey’s, *p* = 0.87). For counts of preening behavior, the best supported model included week, environment complexity, and their interaction (Table S1). Although preening behavior was more common in HC environments compared with LC environments (Figure 2), pairwise comparisons did not provide evidence for differences between individual treatments (Tukey’s, *p* = 0.27, *p* = 0.26, and *p* = 0.99, for week 2, 4, and 7, respectively). The best supported model for the proportion of time spent preening included environmental complexity as a predictor (Table S1). Birds spent a greater proportion of time preening in HC environments compared with LC environments (*p* = 0.03, Table S1, Figure 3).

### 3.3. Foraging

The best supported model for the probability of foraging behavior included environmental complexity, stocking density, age, the interaction between environmental complexity and age, and the interaction between stocking density and age (Table S1). In week 2, there was a negligible effect of stocking density and complexity on the probability of observing foraging behavior (Table 2, Figure 1). In week 4, birds were more likely to be observed foraging in LD/HC treatments compared with HD/LC treatments (Table 2, Figure 1), but this difference was not statistically significant in post hoc pairwise comparisons (Tukey’s, *p* = 0.11). In week 7, birds were more likely to be observed foraging in the LD treatment compared with the HD treatment (Tukey’s, *p* = 0.008 for HC pens, *p* = 0.01 for LC pens). Counts of foraging behavior were unrelated to environmental complexity, stocking density, or week (Table S1). The best supported model for the proportion of time spent foraging included environmental complexity as a predictor (Table S1). Birds spent a greater proportion of time foraging in the HC treatment compared with the LC treatment (*p* = 0.01, Table S1, Figure 3).

### 3.4. Eating

The best supported model for the probability of observing eating included age as a predictor, as well as environmental complexity, stocking density, and their interaction (Table S1). Birds were more likely to be observed eating in week 4 compared with week 2 (*p* = 0.005, Table S1, Figure 1), but no differences were observed between week 2 and week 7 (*p* = 0.19, Table S1), or between week 4 and week 7 (Tukey’s, *p* = 0.24). The probability of observing eating behavior was higher at LD compared with HD in the LC treatment (Tukey’s, *p* = 0.04, Figure 1), but not in the HC treatment (Tukey’s, *p* = 0.76, Figure 1). For counts of eating, the best supported model included stocking density as a predictor with fewer counts of eating at LD compared with the HD treatments (*p* = 0.05, Table S1, Figure 2). The best supported model for the proportion of time spent eating included age as a predictor (Table S1). The proportion of time spent eating was higher in week 2 compared with week 4 (*p* = 0.05, Table S1, Figure 3) and week 7 (*p* = 0.02), but there was no difference in week 4 compared with week 7 (Tukey’s, *p* = 0.78).

### 3.5. Drinking

The best supported model for the probability of observing drinking included age as a predictor with drinking more likely to be observed in week 4 compared with week 2 (*p* = 0.04, Table S1, Figure 1), but there was no difference in week 2 compared with week 7 (*p* = 0.92, Table S1, Figure 1), or between week 4 and week 7 (Tukey’s, *p* = 0.12). For counts of drinking, the best supported model included the effect of age (Table S1). Specifically, counts of drinking behavior were predicted to be higher at week 2 compared with week 4 (*p* < 0.001, Table S1, Figure 2), and was higher at week 7 compared with week 4 (Tukey’s, *p* = 0.005). We found no evidence for differences in counts of drinking behavior between week 2 and week 7 (*p* = 0.85, Table S1, Figure 2). The best supported model for the proportion of time spent drinking included age and stocking density as predictors (Table S1). The proportion of time spent drinking was higher at LD compared with the HD treatments (*p* = 0.03, Table S1, Figure 3). In addition, the proportion of time spent drinking was higher in week 7 compared with week 2 (*p* = 0.05, Table S1, Figure 3) and week 4 (Tukey’s, *p* = 0.04). We found no evidence for a difference in the proportion of time spent drinking between week 2 and week 4 (*p* = 0.69, Table S1, Figure 3).

### 3.6. Locomotion

The best supported model for the probability of observing locomotion included age as a predictor (Table S1). Locomotion was more likely to be observed in week 4 compared with week 2 (*p* < 0.001, Table S1, Figure 1) and week 7 (Tukey’s, *p* = 0.009), and was more likely to be observed in week 7 compared with week 2 (*p* = 0.005, Table S1, Figure 1). For counts of locomotion, the best supported model included stocking density, environmental complexity, age, and their three-way interaction (Table S1). Specifically, counts of locomotion were only higher in HC treatments compared with LC treatments during week 2 at HD (Tukey’s, *p* = 0.02, Figure 2). At weeks 4 and 7, the pattern was reversed, where counts of locomotion were lower in the HC treatments compared with the LC treatments at HD, but these differences were not statistically significant (Tukey’s, week 4: *p* = 0.99, week 7: *p* = 0.97). Across all ages, birds in the HC treatments exhibited fewer counts of locomotion compared with the LC treatments at LD (Figure 2), but in no instance was this difference statistically significant (Tukey’s, week 2: *p* = 0.81, week 4: *p* = 0.81, week 7: *p* = 0.933). The best supported model for the proportion of time spent locomoting included age as a predictor (Table S1). Birds spent a greater proportion of time walking in week 2 compared with week 4 (*p* = 0.05, Table S1, Figure 3) and week 7 (*p* < 0.001, Table S1, Figure 3). Further, birds spent a greater proportion of time walking in week 4 compared with week 7 (Tukey’s, *p* = 0.003).

### 3.7. Additional Behaviors

Perching behavior was more likely to be observed in the HC treatments than the LC treatments (*p* < 0.001, Table S1), but the counts and duration of perching were unrelated to any of the predictors considered. All other behaviors of interest were too rare for a meaningful comparison between treatments. Enrichment interactions were only observed in the HC treatments, totaling fifteen instances across nine birds. Dustbathing was observed in all but the HD/LC treatment, totaling seventeen instances across five birds. Exploratory pecks were observed in all the treatments, totaling 39 instances across 27 birds. Play was observed in all the treatments, totaling 24 instances across 15 birds. All other behaviors not included in the ethogram (Table 1) were observed in all but the LD/HC treatment, totaling 20 instances across 15 birds.

## 4. Discussion

We evaluated the impact of environmental complexity on the behavior of broiler chickens. We found that housing birds in complex environments positively impacted overall activity levels and a range of natural behaviors. However, we also found that the emergent benefits of this complexity diminish as birds age and some benefits can be negated by high stocking densities. Our results shed light on how the affective state of broilers is impacted by housing conditions, and how this knowledge can be leveraged to improve bird welfare.

Broilers were more likely to be active and spent a larger proportion of time being active in HC compared with LC environments. Previous studies have shown that perches [58,59,60], platforms [61], hanging strings [41], and scattering seeds, mealworms, or black soldier fly larvae [62,63] can all result in increased activity levels. However, platforms [64,65] and scattering whole wheat [66,67] showed no impact on broiler behavior, highlighting the need to consider the specific type and variety of enrichments provided [68,69]. Moreover, the impact of environmental complexity on broiler activity was most pronounced in 2-week-old birds. As birds aged, the novelty and quality of the permanent enrichments may have reduced birds’ interest in the objects, as sand got mixed with shavings and fecal material, fecal material may have accumulated on or under perches, and peck stones may have decreased in size. A similar argument was made for sand trays and string enrichments by Arnould et al. [70] and Newberry [28], who observed the reduced attractiveness of novel objects when birds were over 5 weeks old. However, the impact of bird age, the duration of exposure to the same enrichment items (loss of novelty), and the cleanliness of the enrichment items are confounded, and thus cannot be disentangled in the current study. To the authors’ knowledge, no evidence of the impact of cleanliness on enrichment attractiveness is available.

Birds in the HC pens spent more time preening than birds in the LC pens. This was opposite to expectation, as the enrichment items allowed birds to perform behaviors that are not or are less possible in the LC pens, and in turn reduce the time available for preening. For example, perching was almost exclusively seen in the HC pens compared with the LC pens, as the latter treatment did not contain opportunities for perching, besides one side of the pen that consisted of a concrete ledge at approximately 10-cm height. Furthermore, if birds were observed perching, they spent a relatively large proportion of time performing the behavior (up to 80% of the total observed time). In some cases, preening has been interpreted as a displacement behavior indicating possible frustration [7,8,19], however we deem that this is unlikely in the HC pens as highly motivated natural behaviors were positively impacted in the HC treatment compared with the LC treatment, and preening frequency increased with age only in the LC pens. Instead, the increased preening in the HC pens may indicate increased comfort, as comfort behaviors are performed less frequently under stressful conditions [71,72]. Thus, we conclude the higher instance of preening in the complex environments, despite more time being allocated to other behaviors, is a strong indication of positive welfare [73].

Birds in the HC treatment also spent more time foraging than birds in the LC treatment. Counts of foraging behavior did not differ between treatments, however, indicating that foraging bouts were longer in the HC environments compared with the LC environments. The benefits of enrichments on foraging have been shown to depend on the frequency and type of enrichment provided [25,66,67,73]. In the present study, foraging in the HC treatment was disproportionately observed in the space containing the dust bath, whereas foraging in the LC treatments was evenly distributed within the pen. Previous studies have shown broilers exhibiting a preference for sand substrates [74] and increased foraging on sand compared with other substrates ([70] but see [23]). We conclude, therefore, that providing a sand-filled dust bath is an effective method to stimulate foraging behavior in broilers.

In contrast to previous studies [19,23,74], we found no evidence for an effect of environment on dustbathing and play. However, as these behaviors typically only occur at certain times of day [7,75], our study design (birds observed in the morning and afternoon) potentially prevented us from detecting enough instances of play or dustbathing to understand their association with environmental conditions. Repeated scan sampling at key timepoints in the day or longer periods of focal sampling could be beneficial to draw inference about these rare, but important, broiler chicken behaviors.

In addition to the environmental complexity, stocking density had a substantial impact on broiler behavior and, by extension, animal welfare. Bouts of activity in the HD treatment tended to be increased compared with the LD treatments (1454 vs 1261 total bouts, respectively, across all individuals in each treatment), yet birds did not spend more time being active in the HD pens (30% vs. 32% of time observed). This indicates that the durations of active bouts were shorter in HD than LD pens, which suggests that there were more disturbances in HD compared with the LD pens, reflected in an increased frequency of brief movements. Similar effects of stocking density have been shown for a range of poultry ([34,76,77] but see [78]). In the absence of frequent disturbances, and with a greater space provision, birds housed at low densities are able to engage in more natural behaviors. For example, broilers were more likely to be observed foraging in the LD compared with the HD treatment, even at 7 weeks of age. This is in line with previous work documenting a negative impact of high stocking densities on the proportion of birds foraging [79,80]. Similarly, birds spent more time drinking at LD compared with HD and were more likely to be observed eating at LD compared with HD in the LC environment. Such effects of low stocking density on eating and drinking are presumably due to improved access to the feeders and drinkers (less competition with other birds [81]) and the more pronounced differences seen in low complexity pens is likely a result of a lack of stimulation, such that birds simply resort to eating. In support of this claim, eating occurred less frequently at LD compared with HD, yet the proportion of time spent eating was similar, indicating that eating bouts were longer at LD. It is important to note that there was no detrimental effect of HC on eating behavior, regardless of stocking density, even though feeders were not distributed throughout the pen, but rather located in a single concentrated space.

The behavior of broilers, as well as the effects of housing environment on broiler behavior, changed as the birds grew. Surprisingly, more birds were active at 4 and 7 weeks old compared with 2 weeks old. Nearly all the birds observed were active at 4 and 7 weeks, compared with 60–84% of birds at 2 weeks. However, older birds spent a smaller proportion of time active compared with younger birds. This finding is more consistent with previous research reporting broiler chicken activity (time spent active) decreasing as they age (e.g., [19,82,83]).

The continuous lighting during week 2 likely impacted behavior in that week. The proportion of birds being active in week 2 might have been low due to the unintentional, prolonged light period, as lighting regime can impact activity [27,28,29,30]. As active versus passive periods will be less pronounced (all birds will reduce activity in the dark period), it is likely that more passivity during light hours occurred when no dark period was provided.

Age impacted eating and drinking behavior, with more time spent eating at week 2 of age, compared with weeks 4 and 7; this is similar to findings by Jacobs et al. [19] but in contrast with findings by Bizeray et al. [67]. More drinking bouts were observed when birds were younger, which is in line with Jacobs et al. [19], but the proportion of time spent drinking increased as birds aged, which suggests that birds spent more time drinking during a single bout when they were older. This may be due to an increased immobility with age in these fast-growing broilers [84], reflected in a reduced willingness to actively access drinkers repeatedly but rather visit drinkers less often for longer periods of time. However, gait was not assessed, thus this cannot be confirmed.

As birds aged, bouts of preening became more common. Increased preening with age has previously been reported in broilers and argued to be a possible indicator of frustration [7,8,19]. As these fast-growing broilers age, their ability to perform active behaviors is reduced, while their motivation remains similar. This is aligned with the present study and previous findings showing that broiler chickens walk less as they get older [19,74]. Such a conflict between physical ability and motivation could be ‘solved’ by displacement preening, a behavior not expressed to maintain feather condition, but to cope with stressors. For instance, hens will show shorter preening durations and will preen different body parts when in a thwarting situation (stimulating frustration) compared with a control situation [71]. Furthermore, agonistic interactions are interrupted by preening in European starlings, and terns will preen in response to a potential predator [85,86]. Aligned with this hypothesis, broilers in the current study showed shorter preening bouts in week 4 and 7 (approximately 9 s) compared with week 2 (approximately 16 s), while bouts were twice as common. This could be interpreted as a sign of frustration, yet we cannot confirm this based on the results of this study.

Perching frequency and duration were consistent across weeks regardless of stocking density, illustrating the unchanged motivation to perch as broilers age and gain weight, regardless of perch space availability (either in high- or low-density conditions). However, the proportion of birds perching was lower than in previous reports. Similarly designed horizontal PVC perches were used, on average, by 1.9–3.9% of the birds [27,87] compared with less than 1% in the current study. Wooden perches with a flat surface were used by 1–2% of the flock [88]. Differences between the current results and previous findings are likely due to three reasons. One, observations were made during light hours while perching is more highly motivated in the dark (at night), as seen in free-living chickens [89] and laying hens [90], although Norring et al. and Martrenchar et al. [65,91] did not find an effect of the time of day (light versus dark period) on perch use in broilers. Two, perch design might not have met the birds’ physical needs for support of the breast muscle [65]. This is also reflected in broilers’ preference for platforms over perches [92]. Three, the methods of behavioral observations differed, with continuous focal sampling in the current study, rather than instantaneous scan sampling in the other studies [27,87,88].

A limitation of this study was that observers were able to arbitrarily pick birds in the subsection of each space, which could have introduced a minor selection bias. However, we expect this to only have a minimal impact on this study, as birds were selected using a grid-like system with 12 different subsections in the pen. Birds were exposed to a pathogen and were treated at a flock level. It is possible, but not likely, that this impacted behavioral responses, as the week-4 data were collected approximately 7 days prior to disease detection and the week-7 data were collected 5 days after antibiotic treatment ended. The study results indicate that complexity can benefit the expression of natural behaviors. However, it is important to note that here we assessed the impact of ‘complexity’ as a whole, not evaluating the benefits of the specific enrichments separately. Therefore, this limits the potential for the extrapolation of results. What we found is that complexity can benefit natural behaviors under these conditions, but other variations of complexity, e.g., another combination of enrichments, might impact behavior differently.

## 5. Conclusions

With behavior associated with an individual experience, it can be challenging to provide general recommendations to improve production animal welfare [93]. Variation in the effects of different treatments or environments is inevitable. However, here we find that providing a complex environment can stimulate a range of natural active behaviors including foraging, locomotion, preening, and overall activity, confirming our hypotheses. Moreover, despite a reduction in activity levels and a potential sign of frustration over time, many of the benefits of the tested environmental complexity persisted as birds aged. The generally positive impact of both the complexity as tested and the low stocking density on broilers’ behavioral repertoire will ultimately impact their affective state [20,43]. Thus, both more space and a complex environment resulted in a higher level of welfare for fast-growing broiler chickens.

## Figures and Tables

**Table 1:** Ethogram with the behavioral repertoire observed in broiler chickens.

Behavior	Definition
Dustbathing	Bird is lying down, head rubbing, shaking wings vertically, leg scratching, and/or raking the substrate closer to the body with their beak. Bird is clearly lying without performing any other behaviors. End of dustbathing signified by a body-shake.
Preening	Bird runs their beak through their feathers in a seated or standing position. No aspects of dustbathing behavior are observed.
Foraging	Scratching and pecking at the ground from a standing, sitting, or walking position.
Sitting inactive	Sitting down or standing without performing ground pecking or any other behaviors. The bird’s eyes are open and the head is not tucked under a wing.
Locomotion	Walking (taking more than one pace in any direction) or standing with no other activity
Resting	Bird sits with its eyes closed, or with its head beneath one wing/rested on the ground, or the bird lies on one side with or without its eyes closed
Eating	Bird is standing or sitting with its head over the feeder edge
Drinking	Bird is standing or sitting and pecking at drinker
Perching	Bird sits with open or closed eyes on top of the perches.
Exploratory peck	Bird pecks at any object other than feeder, drinker, or litter. This includes pecks at mesh, wood barrier, or concrete barrier.
Enrichment interaction	Bird interacts (pecks, scratches, touches with body) with any of the rotating enrichments. Bird is sitting or standing and pecks/scratches strings, pecking stones, ball, Kong, hay. Bird is sitting or standing. Sitting or standing on the perch excluded.
Play	Spontaneous motor behavior that occurs without apparent purpose. Includes frolicking (sudden running with no apparent stimulus, flapping wings) and food running (object in beak and locomotion at high speed) (adapted from [45])
Other	Any other behavior
Out of view	Bird is out of view of camera

**Table 2:** Proportion of birds (1 would represent 100% of observed birds in each treatment/age category) exhibiting behaviors of interest for each combination of stocking density (HD; high density, LD; low density), environmental complexity (LC; low complexity, HC; high complexity), and age (in weeks). Numbers in parentheses represent the number of bouts (n) of each behavior observed in each treatment.

Age	Stocking Density	Environmental Complexity	Active	Preening	Perching	Foraging	Eating	Drinking	Locomotion
2	HD	LC	0.60 (15)	0.40 (10)	0.00 (0)	0.40 (10)	0.08 (2)	0.16 (4)	0.44 (11)
	LD		0.65 (26)	0.30 (12)	0.03 (1)	0.30 (12)	0.25 (10)	0.25 (10)	0.60 (24)
	HD	HC	0.84 (37)	0.25 (11)	0.30 (13)	0.23 (10)	0.18 (8)	0.25 (11)	0.59 (26)
	LD		0.66 (29)	0.18 (8)	0.25 (11)	0.23 (10)	0.11 (5)	0.14 (6)	0.41 (18)
									
4	HD	LC	0.93 (42)	0.51 (23)	0.02 (1)	0.18 (8)	0.33 (15)	0.38 (17)	0.80 (36)
	LD		0.92 (34)	0.49 (18)	0.00 (0)	0.41 (15)	0.43 (16)	0.30 (11)	0.81 (30)
	HD	HC	1.00 (42)	0.55 (23)	0.29 (12)	0.43 (18)	0.17 (7)	0.21 (9)	0.74 (31)
	LD		1.00 (38)	0.53 (20)	0.18 (7)	0.47 (18)	0.26 (10)	0.32 (12)	0.89 (34)
									
**7**	HD	LC	0.95 (38)	0.58 (23)	0.00 (0)	0.12 (5)	0.17 (7)	0.22 (9)	0.75 (30)
	LD		0.90 (43)	0.35 (17)	0.00 (0)	0.42 (20)	0.27 (13)	0.15 (7)	0.62 (30)
	HD	HC	0.92 (37)	0.52 (21)	0.03 (1)	0.25 (10)	0.30 (12)	0.25 (10)	0.65 (26)
	LD		0.95 (39)	0.51 (21)	0.02 (1)	0.49 (20)	0.15 (6)	0.22 (9)	0.66 (27)

**Table 3:** Mean counts of behaviors of interest for each combination of stocking density (HD; high density, LD; low density), environmental complexity (LC; low complexity, HC; high complexity), and age (in weeks). Means reflect the average count per bird, per five-minute observation period. Numbers in parentheses represent the range of observed counts across all individuals.

Age	Stocking Density	Environmental Complexity	Active	Preening	Perching	Foraging	Eating	Drinking	Locomotion
2	HD	LC	4.73 (1–11)	1.60 (1–3)	0.00	1.90 (1–4)	1.50 (1–2)	2.00 (1–5)	2.27 (1–6)
	LD		7.73 (1–23)	1.75 (1–5)	1.00 (1–1)	2.08 (1–6)	1.20 (1–2)	3.5 (1–7)	4.25 (1–12)
	HD	HC	8.24 (1–57)	2.55 (1–8)	1.62 (1–6)	1.80 (1–4)	2.75 (1–6)	5.18 (1–29)	5.81 (1–26)
	LD		5.28 (1–16)	2.88 (1–8)	1.18 (1–2)	2.50 (1–6)	2.00 (2–2)	2.83 (1–6)	3.50 (1–10)
									
4	HD	LC	7.21 (1–22)	2.39 (1–5)	1.00 (1–1)	1.62 (1–3)	1.93 (1–6)	1.82 (1–5)	4.44 (1–12)
	LD		7.38 (1–16)	1.94 (1–4)	0.00	1.67 (1–3)	1.94 (1–5)	1.91 (1–4)	4.03 (1–10)
	HD	HC	7.83 (1–24)	3.04 (1–8)	1.67 (1–3)	1.61 (1–4)	1.86 (1–4)	2.33 (1–6)	4.55 (1–16)
	LD		6.58 (1–13)	3.00 (1–8)	1.71 (1–5)	1.78 (1–5)	1.40 (1–3)	1.58 (1–2)	2.85 (1–7)
									
7	HD	LC	6.16 (1–16)	2.87 (1–9)	0.00	1.20 (1–2)	3.29 (1–6)	3.89 (1–7)	3.33 (1–12)
	LD		4.88 (1–15)	2.59 (1–6)	0.00	1.85 (1–9)	1.54 (1–4)	3.71 (1–7)	2.20 (1–6)
	HD	HC	5.73 (1–16)	2.81 (1–7)	1 (1–1)	1.70 (1–4)	1.50 (1–3)	3.00 (1–5)	2.85 (1–10)
	LD		5.03 (1–17)	2.19 (1–6)	1 (1–1)	1.85 (1–5)	2.00 (1–6)	4.00 (1–12)	2.11 (1–5)

**Table 4:** Mean proportion of time spent engaged in behaviors of interest for each combination of stocking density (HD; high density, LD; low density), environmental complexity (LC; low complexity, HC; high complexity), and age (in weeks). Means reflect the average time a single bird spent engaged in a behavior during the five-minute observation period and are conditional on the behavior being observed at least once. Numbers in parentheses represent the range of observed proportions across all individuals.

Age	Stocking Density	Environmental Complexity	Active	Preening	Perching	Foraging	Eating	Drinking	Locomotion
2	HD	LC	0.67 (0.01–1.00)	0.21 (0.01–1.00)	0.77 (0.09–1.00)	0.14 (0.01–0.64)	0.40 (0.11–0.97)	0.23 (0.01–0.53)	0.18 (0.01–0.57)
	LD		0.59 (0.02–1.00)	0.15 (0.01–0.59)	0.80 (0.11–1.00)	0.23 (0.06–0.57)	0.34 (0.19–0.53)	0.33 (0.04–0.84)	0.06 (0.01–0.19)
	HD	HC	0.26 (0.01–1.00)	0.08 (0.01–0.32)	0.00	0.04 (0.01–0.13)	0.40 (0.20–0.59)	0.18 (0.05–0.43)	0.10 (0.01–0.68)
	LD		0.40 (0.01–0.99)	0.04 (0.01–0.24)	0.64 (0.64–0.64)	0.07 (0.01–0.21)	0.35 (0.03–0.95)	0.29 (0.07–0.60)	0.09 (0.01–0.31)
									
4	HD	LC	0.37 (0.01–1.00)	0.09 (0.01–0.30)	0.34 (0.04–1.00)	0.14 (0.01–0.97)	0.23 (0.11–0.41)	0.29 (0.05–0.75)	0.08 (0.01–0.29)
	LD		0.49 (0.03–1.00)	0.12 (0.01–0.44)	0.53 (0.07–1.00)	0.19 (0.01–0.91)	0.25 (0.01–0.98)	0.23 (0.03–0.65)	0.07 (0.01–0.33)
	HD	HC	0.23 (0.01–0.85)	0.06 (0.01–0.23)	0.28 (0.28–0.28)	0.04 (0.01–0.20)	0.22 (0.02–0.50)	0.15 (0.05–0.42)	0.05 (0.01–0.15)
	LD		0.40 (0.01–0.92)	0.06 (0.01–0.17)	0.00	0.13 (0.01–0.53)	0.34 (0.06–0.86)	0.25 (0.06–0.69)	0.07 (0.01–0.16)
									
7	HD	LC	0.28 (0.02–0.78)	0.09 (0.01–0.31)	0.06 (0.06–0.06)	0.10 (0.01–0.40)	0.22 (0.10–0.44)	0.35 (0.04–0.78)	0.04 (0.01–0.16)
	LD		0.26 (0.02–0.70)	0.08 (0.01–0.30)	0.38 (0.38–0.38)	0.10 (0.01–0.65)	0.20 (0.01–0.68)	0.37 (0.16–0.67)	0.03 (0.01–0.15)
	HD	HC	0.20 (0.01–0.62)	0.08 (0.01–0.31)	0.00	0.06 (0.01–0.12)	0.24 (0.01–0.45)	0.26 (0.03–0.57)	0.04 (0.01–0.24)
	LD		0.25 (0.01–0.76)	0.09 (0.01–0.21)	0.00	0.10 (0.01–0.65)	0.23 (0.06–0.62)	0.37 (0.16–0.71)	0.03 (0.01–0.12)

## Data Availability

All code needed to replicate our analyses was provided for peer-review and can be found at this Github repository: https://github.com/geobro1992/broiler-welfare.

## Supplementary Materials

The following supporting information can be downloaded at: https://www.mdpi.com/article/10.3390/ani13132074/s1, Table S1: Parameter estimates of the best supported models for counts and duration of chicken behaviors.
