# Supplementary material for: Environmental Complexity and Reduced Stocking Density Promote Positive Behavioral Outcomes in Broiler Chickens
Source: Animals (Basel). 2023 Jun 23;13(13):2074. doi: 10.3390/ani13132074 (PMC10339985; doi:10.3390/ani13132074)
Supplement: Supplementary file 1 [file animals-13-02074-s001.zip › animals-2426438-supplementary.pdf]

Supplementary Table S1. Parameter estimates of the best supported models for counts and duration of chicken behaviors. Predictors included environmental complexity (HC = high complexity), stocking density (LD = low stocking density), week (W), and their interactions. Week 4 (W4) and week 7 (W7) were compared to week 2 as a reference category. Results of post-hoc pairwise comparisons are reported in-text. Significant p-values (≤ 0.05) are bolded.

**Behavior**	**Model for durations**					**Model for nonzero counts**					**Model for zero counts**				
	Estimate	SE	*t*	*p*	Odds	Estimate	SE	*z*	*p*	Odds	Estimate	SE	*z*	*p*	Odds
**Active**															
HC	0.43	0.11	3.86	**<0.001**	1.54	-	-	-	-	-	0.68	0.29	2.33	**0.02**	1.98
LD	-	-	-	-	-	-0.14	0.08	-1.74	0.08	0.87	-	-	-	-	-
W4	-0.34	0.15	-2.27	**0.02**	0.71	0.06	0.10	0.61	0.54	1.06	2.50	0.46	5.48	**<0.001**	12.18
W7	-0.56	0.15	-3.75	**<0.001**	0.57	-0.26	0.10	-2.47	**0.01**	0.77	1.82	0.35	5.15	**<0.001**	6.17
**Preening**															
HC	0.26	0.11	2.16	**0.03**	1.30	0.84	0.37	2.26	**0.02**	2.32	-	-	-	-	-
W4	-	-	-	-	-	0.53	0.34	1.54	0.12	1.69	1.08	0.24	4.48	**<0.001**	2.94
W7	-	-	-	-	-	0.88	0.33	2.63	**0.008**	2.40	0.95	0.24	3.96	**<0.001**	2.57
HC:W4	-	-	-	-	-	-0.36	0.44	-0.82	0.41	0.70	-	-	-	-	-
HC:W7	-	-	-	-	-	-0.98	0.44	-2.26	**0.02**	0.37	-	-	-	-	-
**Perching**															
HC	-	-	-	-	-	-	-	-	-	-	3.25	0.73	4.45	**<0.001**	25.70
**Foraging**															
HC	0.39	0.16	2.52	**0.01**	1.48	-	-	-	-	-	-0.58	0.37	-1.57	0.11	0.56
LD	-	-	-	-	-	-	-	-	-	-	-0.21	0.37	-0.57	0.57	0.81
W4	-	-	-	-	-	-	-	-	-	-	-0.69	0.46	-1.52	0.13	0.50
W7	-	-	-	-	-	-	-	-	-	-	-1.18	0.49	-2.41	**0.02**	0.31
HC:W4	-	-	-	-	-	-	-	-	-	-	1.32	0.50	2.65	**0.008**	3.75
HC:W7	-	-	-	-	-	-	-	-	-	-	1.06	0.51	2.10	**0.04**	2.89
LD:W4	-	-	-	-	-	-	-	-	-	-	0.82	0.50	1.65	0.10	2.28
LD:W7	-	-	-	-	-	-	-	-	-	-	1.51	0.52	2.92	**0.004**	4.52
**Eating**															
HC	-	-	-	-	-	-	-	-	-	-	0.05	0.32	0.16	0.87	1.05
LD	-	-	-	-	-	-0.53	0.27	-1.97	**0.05**	0.59	0.58	0.31	1.89	**0.05**	1.78
W4	-0.47	0.25	-1.90	**0.05**	0.63	-	-	-	-	-	0.79	0.28	2.81	**0.005**	2.21
W7	-0.62	0.26	-2.39	**0.02**	0.54	-	-	-	-	-	0.38	0.29	1.32	0.19	1.46
HC:LD	-	-	-	-	-	-	-	-	-	-	-0.85	0.45	-1.90	**0.05**	0.43

**Drinking**															
LD	0.35	0.16	2.16	**0.03**	1.41	-	-	-	-	-	-	-	-	-	-
W4	-0.08	0.20	-0.40	0.69	0.92	-1.14	0.29	-3.93	**<0.001**	0.32	0.53	0.26	2.02	**0.04**	1.71
W7	0.40	0.21	1.91	**0.05**	1.49	-0.05	0.28	-0.19	0.85	0.95	0.03	0.28	0.10	0.92	1.03
**Locomotion**															
HC	-	-	-	-	-	1.25	0.39	3.22	**0.002**	3.48	-	-	-	-	-
LD	-	-	-	-	-	0.88	0.39	2.23	**0.03**	2.41	-	-	-	-	-
W4	-0.23	0.11	-1.98	**0.05**	0.80	0.93	0.38	2.46	**0.01**	2.54	1.38	0.26	5.35	**<0.001**	3.96
W7	-0.60	0.12	-4.92	**<0.001**	0.55	0.57	0.39	1.46	0.15	1.77	0.64	0.23	2.78	**0.005**	1.89
HC:W4	-	-	-	-	-	-1.22	0.45	-2.74	**0.01**	0.30	-	-	-	-	-
HC:W7	-	-	-	-	-	-1.47	0.47	-3.10	**0.002**	0.23	-	-	-	-	-
LD:W4	-	-	-	-	-	-1.00	0.46	-2.19	**0.03**	0.37	-	-	-	-	-
LD:W7	-	-	-	-	-	-1.50	0.48	-3.11	**0.002**	0.22	-	-	-	-	-
HC:LD	-	-	-	-	-	-1.49	0.49	-3.07	**0.002**	0.23	-	-	-	-	-
HC:LD:W4	-	-	-	-	-	1.00	0.59	1.71	0.09	2.72	-	-	-	-	-
HC:LD:W7	-	-	-	-	-	1.64	0.64	2.56	**0.01**	5.14	-	-	-	-	-
**Inactive**															
HC	-	-	-	-	-	0.63	0.20	3.06	**0.002**	1.87	-3.24	1.03	-3.15	**0.002**	0.04
LD	-	-	-	-	-	0.36	0.25	1.48	0.14	1.44	-	-	-	-	-
W4	-	-	-	-	-	0.95	0.23	4.11	**<0.001**	2.59	1.35	0.49	2.75	**0.006**	3.86
W7	-	-	-	-	-	1.34	0.23	5.82	**<0.001**	3.82	NA	NA	NA	NA	NA
HC:W4	-	-	-	-	-	-0.58	0.29	-1.98	**0.05**	0.56	-	-	-	-	-
HC:W7	-	-	-	-	-	-0.64	0.29	-2.22	**0.03**	0.53	-	-	-	-	-
LD:W4	-	-	-	-	-	-0.48	0.30	-1.62	0.11	0.62	-	-	-	-	-
LD:W7	-	-	-	-	-	-0.84	0.29	-2.90	**0.004**	0.43	-	-	-	-	-
HC:LD	-	-	-	-	-	-0.97	0.33	-2.94	**0.003**	0.38	-	-	-	-	-
HC:LD:W4	-	-	-	-	-	0.90	0.41	2.22	**0.03**	2.47	-	-	-	-	-
HC:LD:W7	-	-	-	-	-	1.14	0.39	2.90	**0.004**	3.13	-	-	-	-	-
